# The Influence of Oral Bacteria on Epithelial Cell Migration *In Vitro*

**DOI:** 10.1155/2013/154532

**Published:** 2013-10-30

**Authors:** Alexa M. G. A. Laheij, Johannes J. de Soet, Enno C. I. Veerman, Jan G. M. Bolscher, Cor van Loveren

**Affiliations:** ^1^Department of Preventive Dentistry, Academic Centre for Dentistry Amsterdam (ACTA), University of Amsterdam and VU University Amsterdam, Gustav Mahlerlaan 3004, 1081 LA Amsterdam, The Netherlands; ^2^Department of Oral Biochemistry, Academic Centre for Dentistry Amsterdam (ACTA), University of Amsterdam and VU University Amsterdam, Gustav Mahlerlaan 3004, 1081 LA Amsterdam, The Netherlands

## Abstract

Oral ulcerations often arise as a side effect from chemo- and radiation therapy. In a previous clinical study, *Porphyromonas gingivalis* was identified as a positive predictor for oral ulcerations after hematopoetic stem cell transplantation, possibly incriminating *P. gingivalis* in delayed healing of the ulcerations. Therefore, it was tested whether *P. gingivalis* and its secreted products could inhibit the migration of oral epithelial cells in an *in vitro* scratch assay. To compare, the oral bacteria *Prevotella nigrescens*, *Prevotella intermedia*, *Tannerella forsythia*, and *Streptococcus mitis* were included. A standardized scratch was made in a confluent layer of human oral epithelial cells. The epithelial cells were challenged with bacterial cells and with medium containing secretions of these bacteria. Closure of the scratch was measured after 17 h using a phase contrast microscope. *P. gingivalis*, *P. nigrescens*, and secretions of *P. gingivalis* strongly inhibited cell migration. A challenge with 1000 heat-killed bacteria versus 1 epithelial cell resulted in a relative closure of the scratch of 25% for *P. gingivalis* and 20% for *P. nigrescens*. Weaker inhibitory effects were found for the other bacteria. The results confirmed our hypothesis that the oral bacteria may be involved in delayed wound healing.

## 1. Introduction

The oral mucosa serves as a physical barrier to protect the underlying tissues from the entry of microorganisms from the oral cavity. The outer layer of the oral mucosa consists of epithelial cells [1] that are in constant contact with these oral microorganisms. Loss of integrity of the physical barrier, as in the case of ulceration, can lead to infectious complications such as bacteraemia and sepsis [2–4]. Ulcerations of the oral mucosa often occur as a side effect of chemo- and radiation therapy for cancer treatment [5, 6]. These ulcerations are very painful and cause the patient substantial discomfort. Ulceration is an advanced stage of mucositis that has a complex pathobiology consisting of several consecutive stages, initiation, primary damage response, signal amplification, ulceration, and healing [2]. Healing consists of three phases that overlap in time, inflammation, cell proliferation and reepithelialisation, and remodeling of the tissue [7]. Cell migration is a major step in the reepithelialisation process. To study cell migration, *in vitro* scratch assays are commonly used with the scratched area before and after migration as the main outcome parameter [8]. 

The role that microorganisms play in maintaining and healing oral ulcerations after chemo- and radiation therapy is not yet clear [9]. Microorganisms are thought to intensify the inflammatory response and to further damage the mucosa [2]. In a previous prospective clinical study, we studied the relationship between bacteria that are associated with periodontitis and oral ulcerations in hematopoietic stem cell transplant (HSCT) recipients [10]. Periodontal pathogens were selected in these experiments because they demonstrated tissue damaging effects in periodontitis patients [11]. We found that the Gram-negative anaerobic bacterium *Porphyromonas gingivalis* was a positive predictor for the presence of oral ulcerations after HSCT. *Parvimonas micra* and *Fusobacterium nucleatum* possibly influenced oral ulcerations; however *Prevotella intermedia* and *Tannerella forsythia* were not predictors of oral ulcerations [10]. 

In the current study, we explored the hypothesis that *P. gingivalis *and its secreted products inhibit wound closure, causing delayed healing of oral ulcerations. We examined the effect of *P. gingivalis *on the migration of oral epithelial cells in an *in vitro *scratch assay. To compare, we included *P. intermedia, T. forsythia*, the bacteria which emerged from our previous study [10], *P. nigrescens*, which is closely related to *P. intermedia* and is associated with “healthy” supragingival plaque [12], and *Streptococcus mitis*, which is associated with oral health [13].

## 2. Materials and Methods

### 2.1. Epithelial Cells

The human buccal epithelial cell line HO-1-N-1 was provided by the Japanese Collection of Research Bioresources (Osaka, Japan). The cells were cultured in DMEM-F12 medium (Invitrogen, Carlsbad, CA, USA) supplemented with 10% fetal calf serum (Hyclone, Logan, UT, USA), 100 U/mL penicillin, 100 *μ*g/mL streptomycin, and 250 ng/mL amphotericin B (all from Sigma, St. Louis, MO, USA) in a humidified atmosphere with 5% CO_2_ at 37°C. Cells were grown until confluence, detached with 0.25% trypsin-EDTA (Invitrogen), counted with a hemacytometer, and seeded in 24-well plates at cell densities of 3–5·10^5^ cells/mL in DMEM-F12 medium.

### 2.2. Bacterial Strains and Culture

*P. gingivalis* W83 was cultured in Brain-Heart-Infusion (BHI; BD Difco, Le Pont de Claix, France) broth enriched with hemin (5 mg/L) and menadione (1 mg/L). *P. intermedia* ATCC 25611 and *P. nigrescens* ATCC 33563 were cultured in Tryptic-Soy Broth (BD Difco) supplemented with hemin (5 mg/L), menadione (1 mg/L), and glucose (56 nM). *T. forsythia *ATCC 43037 was cultured in BHI broth (39 g/L) supplemented with yeast extract (1 g/L), fetal calf serum (10%) (HyClone), hemin (5 mg/L), menadione (500 *μ*g/L), cysteine (1 g/L), and N-acetylmuramic acid (1.5 mL/L). *S. mitis* LMG 14557 was cultured in BHI broth. *P. gingivalis*, *P. intermedia*, *P. nigrescens*, and *T. forsythia* were cultured anaerobically (80% N_2_, 10% H_2_, and 10% CO_2_), and *S. mitis* was grown aerobically at 37°C until log-phase growth. Bacterial cultures were checked for purity by culturing and Gram staining. 

### 2.3. Viable and Heat-Killed Bacteria

*P. gingivalis* viable cultures used for the scratch assay were washed twice with Dulbecco's DPBS (Invitrogen) and resuspended in keratinocyte serum-free medium (SFM, Invitrogen) at the required OD_690_. An OD_690_ of 0.1 corresponded to 5·10^8^ CFU/mL. In every scratch assay, a freshly prepared bacterial culture was used.

*P. gingivalis*, *P. intermedia*, *P. nigrescens*, and *T. forsythia *were killed by incubation of the bacterial cultures at 60°C for 60 min. *S. mitis* was killed by incubation of the culture at 80°C for 10 min. Killing was confirmed by absence of growth on blood agar plates. After these treatments, the cell wall of the bacteria remained intact, as was confirmed by Gram staining. After killing, the bacteria were washed twice with Dulbecco's DPBS, resuspended in SFM at the required OD_690_, and stored at −80°C until use in the scratch assays.

### 2.4. Preparation of Conditioned Medium

Conditioned medium was prepared as described before [14]. *P. gingivalis*, *P. nigrescens*, and* S. mitis* were grown until log phase as described above. Bacteria were washed twice with DPBS and resuspended in SFM at the required OD_690_. Cultures were incubated anaerobically for *P. gingivalis*, *P. nigrescens*, and aerobically for *S. mitis* at 37°C for 6 h. Bacteria were removed from the medium by centrifugation, and the supernatant (conditioned medium) was filter sterilized (0.2 *μ*m) (Sarstedt, Nümbrecht, Germany) and stored at −80°C until use. Conditioned medium contained secreted metabolites, outer membrane vesicles, proteolytic enzymes, and signaling molecules of the bacteria [15, 16].

To prevent *S. mitis* from growing out in SFM, the antibiotic tetracycline was added at sub-MIC concentrations (5 mg/L). Tetracycline inhibits protein synthesis and thereby bacterial growth [17]. After incubation with *S. mitis*, SFM became depleted of glucose, highlighting active glucose metabolism by the bacteria [18]. In line, the pH decreased from 7.3 to 6.8. Both the glucose and pH level were restored to the original values (6.6 mM and pH 7.3) before use in the experiments. In the control SFM tetracycline was added. For every wound closure experiment, a freshly prepared batch of conditioned medium was used.

### 2.5. Wound Closure Assay

Scratch assays were performed as previously described with slight modifications [19]. Cells were seeded in 24 well plates at 3–5·10^5^ cells/mL in DMEM-F12 and grown until confluence. Cells were washed with DPBS. In each well, a scratch was made with the tip of a sterile blue pipet point (Greiner Bio-One, Alphen a/d Rijn, The Netherlands). Cells were washed twice with DPBS to remove detached cells. In each well, live or heat-killed bacteria, or conditioned medium was added. The scratch was photographed immediately and after 17 h with an inverted digital phase contrast microscope EVOS FL (Advanced Microscopy Group, USA), and the surface of the scratch was calculated with Photoshop CS4 (version 11.0.1, Adobe).

Closure percentage of the scratch was calculated as 100 − ((surface of the scratch at time 17 h/surface of the scratch at time 0) ∗ 100). Relative closure was calculated as the percentage of closure of the treatment/percentage of closure of the control (SFM). The closure of the scratch under control conditions was 1. Each treatment was performed in quintuple, and each experiment was completed on three separate occasions.

### 2.6. Live/Dead Staining

A PromoKine live/dead stain Kit II for cells (PromoCell GmbH, Heidelberg, Germany) was performed to ensure epithelial cell viability after infection with *P. gingivalis*. Cells were washed with DPBS. 1 mL of live/dead stain with a 2 *μ*M calcein AM and 4 *μ*M EthD-III per well was added and incubated at room temperature for 30 min. Cell viability was observed with an EVOS FL microscope; viable cells fluoresced brightly.

### 2.7. Statistical Analysis

Results of three separate experiments with the same conditions were pooled. Differences in relative closure of the scratch between bacteria and bacterial products versus control were tested with the nonparametric Mann-Whitney *U* test. Statistical analyses were performed in SPSS version 20.0 (IBM SPSS, Chicago, IL, USA). A *P* value < 0.05 was considered statistically significant.

## 3. Results

**Figure 1:**
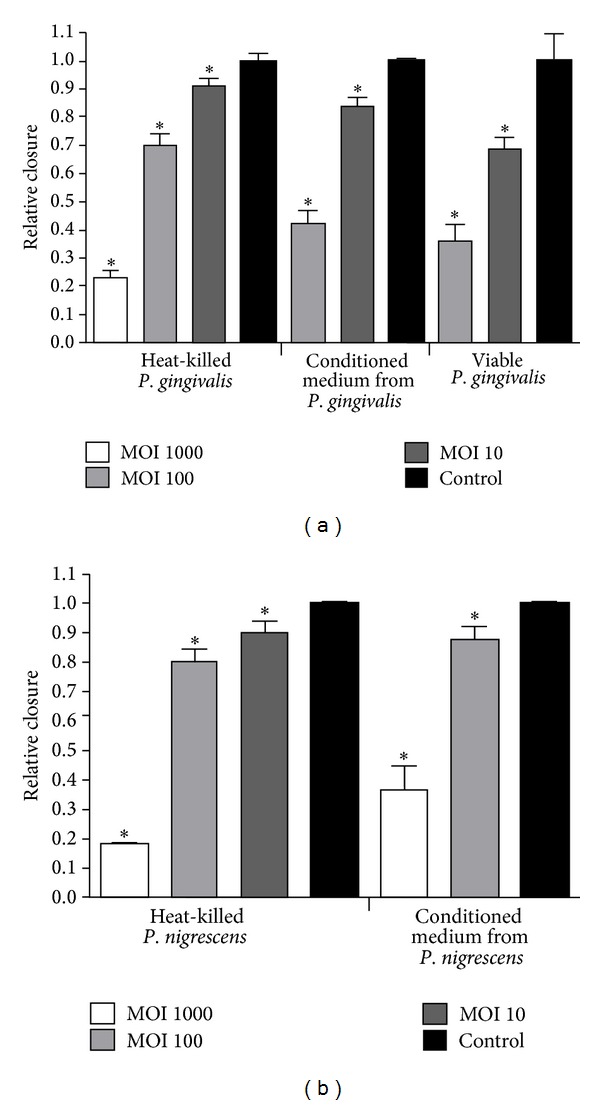
Relative closure (mean + SEM) of scratch in oral epithelial cells challenged with (a) different concentrations of heat inactivated *P. gingivalis*, conditioned medium from *P. gingivalis*, and viable *P. gingivalis.* Relative closure significantly different (*P* < 0.05) from control is marked with ∗ (b) different concentrations of heat inactivated *P. nigrescens* and conditioned medium from *P. nigrescens*. Relative closure significantly different (*P* < 0.05) from control is marked with ∗.

**Figure 2:**
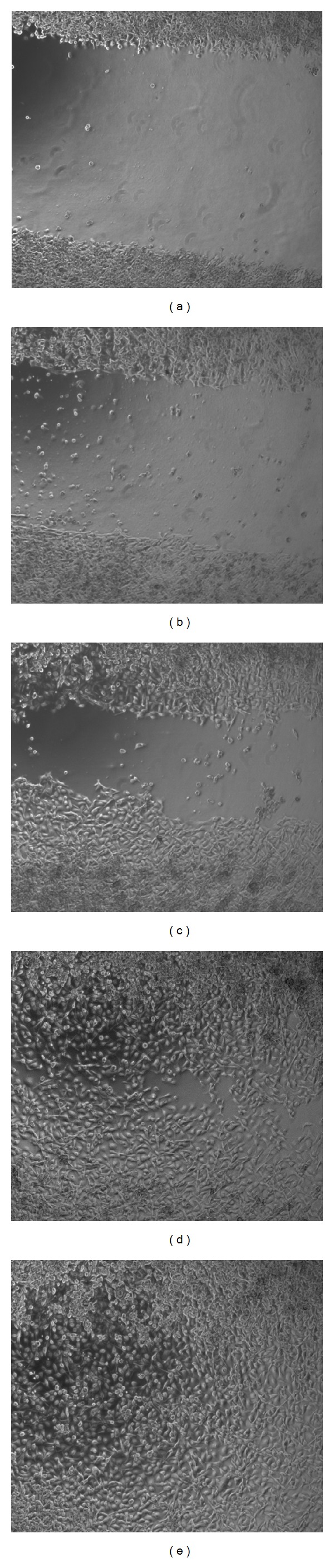
Representative micrographs of a challenge with different numbers of heat-killed *P. gingivalis* and control medium. (a) Original scratch (b)–(e) after 17 h incubation, (b) MOI 1000 heat-killed *P. gingivalis*, (c) MOI 100 heat-killed *P. gingivalis*, (d) MOI 10 heat-killed *P. gingivalis*, and (e) control medium.

The inhibition of oral epithelial cell migration by *P. gingivalis* is shown in Figures 1(a) and 2. In the presence of 1000 heat-killed *P. gingivalis *versus 1 epithelial cell (multiplicity of infection (MOI) of 1000) closure of the scratch relative to control conditions where the closure varied from 80 to 100% of the initial scratch, was approximately 25%. At MOI 100, relative closure of the scratch was about 70%, and at MOI 10 it was about 90%. For conditioned medium from *P. gingivalis* and viable *P. gingivalis*, detachment of epithelial cells from the bottom of the well was observed at the highest MOI tested (1000 bacteria/cell). The inhibition of relative closure of the scratch was higher when challenged with conditioned medium from *P. gingivalis *and viable *P. gingivalis* compared to when challenged with heat-killed bacteria at an MOI of 100 (*P* < 0.05). At MOI 10, conditioned medium and especially viable *P. gingivalis* produced an inhibition comparable to that of a tenfold higher number (MOI 100) of heat-killed *P. gingivalis*.

**Figure 3:**
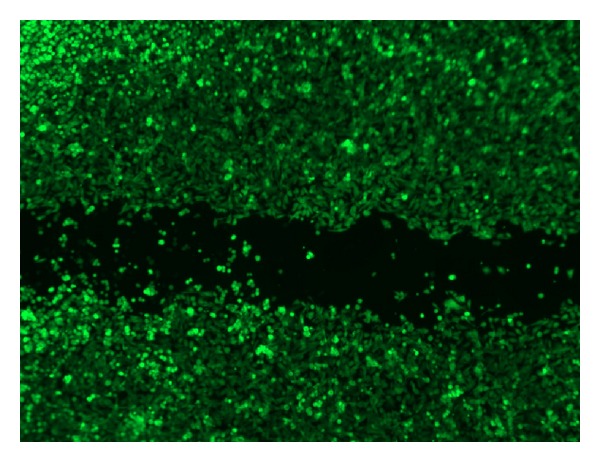
Representative example of live/dead staining of oral epithelial cells after 17 h challenge with heat inactivated *P. gingivalis* and control medium.

To examine if *P. gingivalis* adversely affected the viability of the epithelial cells, a live/dead staining was conducted (Figure 3). All the adhered epithelial cells exhibited a green fluorescence, indicating that their viability was maintained when they were challenged with *P. gingivalis* at MOI 1000. Moreover, the epithelial cells remained adhered to the well, they had a normal morphology, and cells around the scratch were stretching, confirming cell viability as well.

Heat-killed *P. nigrescens *inhibited cell migration at high MOI of 1000 to the same extent as *P. gingivalis;* relative closure was for approximately 20%. Conditioned medium from *P. nigrescens* was less effective in inhibiting cell migration than heat inactivated bacteria (*P* < 0.05, Figure 1(b)). 

**Figure 4:**
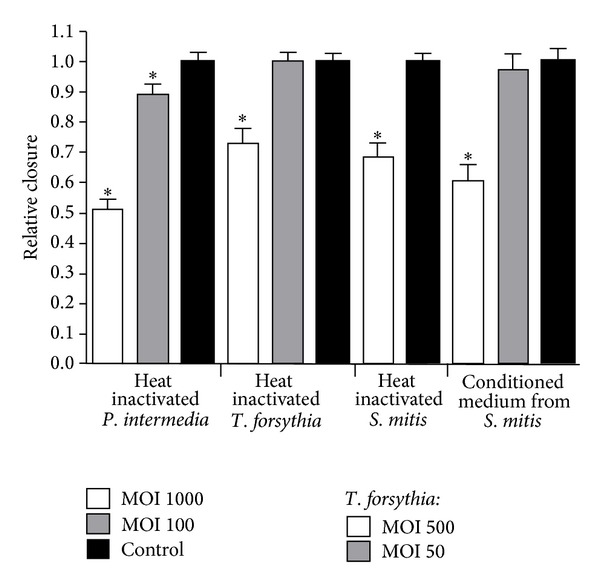
Relative closure (mean + SEM) of scratch in oral epithelial cells challenged with different numbers of heat inactivated *P. intermedia*, *T. forsythia*, *S. mitis*, and conditioned medium from *S. mitis*. Relative closure significantly different (*P* < 0.05) from control is marked with ∗.

Weaker inhibitory effects were found for heat-killed *P. intermedia*,* T. forsythia*, *S. mitis*, and conditioned medium from *S. mitis* (Figure 4). At the highest bacteria/cell ratio of heat-killed *P. intermedia*, relative closure was approximately 50%, and for *T. forsythia*, *S. mitis*, and conditioned medium from *S. mitis*, relative closure was between 60 and 75%. Heat-killed *S. mitis *attached firmly to the bottom of the well and formed a viscous layer in the remaining scratch after 17 h incubation. Despite the presence of this layer on the bottom of the well, relative closure was about 70%, and the rate of cell migration did not seem to be affected. 

Conditioned medium from *P*. *intermedia *and *T. forsythia* could not be made, and experiments with viable *P. nigrescens, P*. *intermedia*,* T. forsythia*, and *S. mitis* could not be performed due to specific needs for growth conditions. 

## 4. Discussion

The aim of this study was to explore the influence of the oral bacteria *P. gingivalis, P. intermedia*,* P. nigrescens*, *T. forsythia*, and *S. mitis* on the migration of oral epithelial cells in an *in vitro *scratch assay as a model for wound healing. *P. gingivalis *and *P. nigrescens* significantly inhibited the cell migration of oral epithelial cells. This finding supports our hypothesis that *P. gingivalis* may be involved in the delayed healing of oral ulcerations after HSCT. *P. nigrescens* inhibited cell migration unexpectedly. Because this bacterium is associated with plaque in a periodontally healthy situation [12], we did not anticipate such an inhibitory effect. *P. intermedia*, *T. forsythia*, and *S. mitis* had the lowest effects, as expected from the results of our clinical study where they were not associated with oral ulcerations [10]. 

The results for *P. gingivalis *are in line with two other studies that described the inhibition of cell migration by viable *P. gingivalis *[20, 21]. To our knowledge, we are the first to report an inhibiting effect of different concentrations of heat-killed *P. gingivalis* and conditioned medium from *P. gingivalis*. Moreover, this is the only study that demonstrates the inhibition of epithelial cell migration by *P. nigrescens*, *P. intermedia*, *T. forsythia*, and *S. mitis*.

 Some mechanisms of inhibition of cell migration may be excluded in our experiments, that is, epithelial cell death and physical hindrance by the bacteria occupying the scratched area. Epithelial cell viability was confirmed by live/dead staining and from the morphology (stretching) of the epithelial cells. After a challenge with the highest MOI of viable *P. gingivalis* dead and detached epithelial cells were seen. After a challenge with the other MOI's of viable *P. gingivalis* and all other conditions tested no dead and detached epithelial cells were seen. If physical hindrance by bacteria would have played an important role in inhibiting cell migration, we would have expected a strong effect of *S. mitis*, since it is very hydrophobic [22] and the bacterial cells precipitated as a sticky layer on the bottom of the well in the scratch. However, *S. mitis *did not inhibit epithelial cell migration effectively, and therefore, we conclude that, at least for *S. mitis,* physical hindrance does not play an important role in inhibiting cell migration.

The factors that are responsible for the inhibiting effect of *P. gingivalis* on cell migration are associated with the cell wall and are actively secreted by the bacterium. It has been reported that parts of the cell wall of *P. gingivalis* are shed as outer membrane vesicles, and therefore, the inhibiting factor in both the cellular fraction and the conditioned medium can comprise cell wall associated compounds [23]. A good candidate cell wall structure both in intact cells and in the outer membrane vesicles in Gram-negative bacteria responsible for the inhibition of cell migration is lipopolysaccharide (LPS) because of its known effects on inflammatory cytokines and bone resorption and its heat resistance [14, 24, 25]. Other virulence factors of *P. gingivalis* that are good candidates for the inhibiting effect are the cell wall-bound fimbriae and the capsular polysaccharide [26, 27]. Heat-labile factors are responsible for an additional effect of conditioned medium and viable bacteria compared to heat-killed bacteria. LPS, the fimbriae, and the capsular polysaccharide are quite heat stable. On the other hand, the proteases that *P. gingivalis* secretes are heat labile, and therefore, this important virulence factor of *P. gingivalis *may be responsible for that additional effect.

 In *P. nigrescens*, where heat-killed bacteria inhibit cell migration more than conditioned medium, LPS is probably the most important factor that causes the inhibition of cell migration. In conditioned medium from *P. nigrescens *proteases and cytotoxic end products like succinate, acetate, and ammonium [28] are present which might all inhibit cell migration. The production of outer membrane vesicles has not been reported for this bacterium. In the other tested bacteria, the causal factors are most likely present in or on the cell wall.

## 5. Conclusions

*P. gingivalis* and *P. nigrescens* and secreted products of *P. gingivalis* strongly inhibited migration of oral epithelial cells in an *in vitro* scratch assay. The other tested bacteria inhibited wound closure as well however to a lesser extent.
